# 2060 Exploring gene expression signature shared between obese Zucker rat and human cardiac hypertrophy

**DOI:** 10.1017/cts.2018.71

**Published:** 2018-11-21

**Authors:** Mackenzie Newman, Janelle Stricker, Han-Gang Yu

**Affiliations:** Department of Physiology, Pharmacology, and Neuroscience, West Virginia University, Morgantown, WV, USA

## Abstract

OBJECTIVES/SPECIFIC AIMS: Objectives: To determine genes that are shared between human and obese Zucker rat hypertrophic hearts, in order to identify potential early biomarkers and drug target for heart failure. METHODS/STUDY POPULATION: Four age-paired lean and obese Zucker rats were used. The human data are derived from doi:10.1152/physiolgenomics.00122.2016. RESULTS/ANTICIPATED RESULTS: We expect to find genes that are upregulated and downregulated in Zucker rats and humans that present cardiac hypertrophy. DISCUSSION/SIGNIFICANCE OF IMPACT: The genes and proteins determined from this study will provide future directions in order to determine whether obese Zucker rats are a valid model organism for the development of cardiac hypertrophy.

